# The variability and reproducibility of whole genome sequencing technology for detecting resistance to anti-tuberculous drugs

**DOI:** 10.1186/s13073-016-0385-x

**Published:** 2016-12-22

**Authors:** Jody Phelan, Denise M. O’Sullivan, Diana Machado, Jorge Ramos, Alexandra S. Whale, Justin O’Grady, Keertan Dheda, Susana Campino, Ruth McNerney, Miguel Viveiros, Jim F. Huggett, Taane G. Clark

**Affiliations:** 1 Department of Pathogen Molecular Biology, Faculty of Infectious and Tropical Diseases, London School of Hygiene & Tropical Medicine, Keppel Street, WC1E 7HT London, UK; 2 Molecular Biology, LGC Ltd, Queens Road, Teddington, Middlesex TW11 0LY UK; 3 Unidade de Microbiologia Médica, Global Health and Tropical Medicine, GHTM, Instituto de Higiene e Medicina Tropical, IHMT, Universidade NOVA de Lisboa, UNL, Lisbon, Portugal; 4 Norwich Medical School, University of East Anglia, Norwich Research Park, Norwich, NR4 7TJ UK; 5 Division of Pulmonary Medicine and UCT Lung Institute, Lung Infection and Immunity Unit, University of Cape Town, Groote Schuur Hospital, Observatory, 7925, Cape Town, South Africa; 6 School of Biosciences & Medicine, Faculty of Health & Medical Science, University of Surrey, Guildford, GU2 7XH UK; 7 Faculty of Epidemiology and Population Health, London School of Hygiene & Tropical Medicine, WC1E 7HT London, UK

**Keywords:** Drug resistance, Tuberculosis, Diagnostics, Drug-susceptibility testing, XDR-TB, Next-generation sequencing

## Abstract

**Background:**

The emergence of resistance to anti-tuberculosis drugs is a serious and growing threat to public health. Next-generation sequencing is rapidly gaining traction as a diagnostic tool for investigating drug resistance in *Mycobacterium tuberculosis* to aid treatment decisions. However, there are few little data regarding the precision of such sequencing for assigning resistance profiles.

**Methods:**

We investigated two sequencing platforms (Illumina MiSeq, Ion Torrent PGM™) and two rapid analytic pipelines (*TBProfiler*, *Mykrobe predictor*) using a well characterised reference strain (H37Rv) and clinical isolates from patients with tuberculosis resistant to up to 13 drugs. Results were compared to phenotypic drug susceptibility testing. To assess analytical robustness individual DNA samples were subjected to repeated sequencing.

**Results:**

The MiSeq and Ion PGM systems accurately predicted drug-resistance profiles and there was high reproducibility between biological and technical sample replicates. Estimated variant error rates were low (MiSeq 1 per 77 kbp, Ion PGM 1 per 41 kbp) and genomic coverage high (MiSeq 51-fold, Ion PGM 53-fold). MiSeq provided superior coverage in GC-rich regions, which translated into incremental detection of putative genotypic drug-specific resistance, including for resistance to para-aminosalicylic acid and pyrazinamide. The *TBProfiler* bioinformatics pipeline was concordant with reported phenotypic susceptibility for all drugs tested except pyrazinamide and para-aminosalicylic acid, with an overall concordance of 95.3%. When using the *Mykrobe predictor* concordance with phenotypic testing was 73.6%.

**Conclusions:**

We have demonstrated high comparative reproducibility of two sequencing platforms, and high predictive ability of the *TBProfiler* mutation library and analytical pipeline, when profiling resistance to first- and second-line anti-tuberculosis drugs. However, platform-specific variability in coverage of some genome regions may have implications for predicting resistance to specific drugs. These findings may have implications for future clinical practice and thus deserve further scrutiny, set within larger studies and using updated mutation libraries.

**Electronic supplementary material:**

The online version of this article (doi:10.1186/s13073-016-0385-x) contains supplementary material, which is available to authorized users.

## Background

*Mycobacterium tuberculosis*, the bacterium that causes tuberculosis disease (TB), has overtaken HIV as the world’s major cause of death from an infectious agent [1]. In recent years, control of the disease has been made more difficult by the emergence of multidrug-resistant tuberculosis (MDR-TB), which is resistant to at least rifampicin and isoniazid, and extensively drug-resistant (XDR-TB), which refers to additional resistance to the fluoroquinolones and second-line injectable drugs (amikacin, kanamycin and capreomycin) used to treat MDR-TB [2]. Programmatically incurable TB with resistance to up to 14 drugs has been reported in several parts of the world, including countries with a high TB burden such as India and South Africa [3, 4]. Phenotypic methods of determining susceptibility to anti-TB drugs take weeks or months, they are additively costly, and require culture and manipulation of large numbers of highly infectious bacilli. Drug resistance in *M. tuberculosis* is almost exclusively due to mutations in the circular genome and so molecular determination of resistance offers a rapid, potentially cost effective, and safer alternative. Commercially available molecular-based tests and line probe assays cover a limited number of drugs but, with the exception of rifampicin, they have relatively low sensitivity for detecting all possible molecular targets for resistance [5]. Due to the multiplicity of drugs used in the treatment of TB, determining the full resistance profile for a patient suspected of having drug-resistant disease requires the examination of many loci.

Next-generation whole genome sequencing offers an attractive option as it simultaneously examines all loci and provides information regarding both small and large changes in the genome [5]. This option has been widely reported as a means of identifying putative resistance-causing mutations and more recently has been used in the management of patients with drug-resistant TB to guide selection of appropriate drug regimens [6–11]. This approach is significant because the current treatment outcomes for MDR-TB are poor, largely due to current molecular tests being unable to guide effective individualised therapy. It also has public health implications because of prolonged patient infectiousness due to suboptimal treatment.

The *M. tuberculosis* genome is challenging to sequence due to its high GC content and repetitive nature. Surprisingly, despite the serious consequences of misdiagnosis, there is a paucity of data regarding the reliability of next-generation sequencing platforms or the analytical methodology used for assigning resistance [5]. To address this issue we investigated the utility of two commercial sequencing platforms for predicting resistance to 13 anti-TB drugs. We also examined analytical algorithms and two rapid bioinformatics tools (*TBProfiler*, *Mykrobe predictor*) for predicting resistance from raw sequence data. Testing was performed with a fully susceptible reference strain (H37Rv) and ten clinical isolates from patients with drug-resistant TB.

## Methods

### Samples

*M. tuberculosis* clinical isolates were sourced from ten patients with known drug-resistant TB admitted to four different hospitals in Lisbon between 2007 and 2013. These samples were not part of a transmission chain and there is no epidemiological link between the patients. All clinical samples and the reference strain H37Rv (ATCC 25618D-9, Lot # 60986340) were prepared by inoculating a single colony into Middlebrook 7H9 broth supplemented with 10% OADC (Becton Dickinson) (see Table 1 for list). Susceptibility testing for the first-line anti-TB drugs rifampicin (RIF), isoniazid (INH), ethambutol (ETB), pyrazinamide (PZA) and streptomycin (STR) and the second-line drugs rifabutin (RFB), amikacin (AMK), capreomycin (CAP), ofloxacin (OFX), moxifloxacin (MOX), ethionamide (ETH), para-aminosalicylic acid (PAS) and linezolid (LZ) was performed on all strains with the MGIT960 system (Becton Dickinson), according to the manufacturer’s instructions. Quantitative drug susceptibility testing (qDST) for both first- and second-line drugs was conducted using a combination of the MGIT960 system and the Epicenter V5.80A software equipped with the TB eXIST module (Becton Dickinson) [12, 13].

**Table 1:** Study samples (DNA extracted from culture isolates) and their susceptibility to anti-tuberculosis drugs

Sample	Year^a^	Lineage	Spoligo. family	Drug susceptibility test phenotype														
				INH	RIF	STR	ETB	PZA	RFB	ETH	AMK	CAP	OFX	MOX	PAS	LZ	KAN^b^	Resistance phenotype
POR1	2007	4.3.4.2	LAM4	R	R	**R**	R	R	R	R	R	R	R	R	R	S	R	XDR-TB
POR2	2007	4.1.1.1	X2	**R**	R	S	S	S	R	R	S	S	S	S	S	S	-	MDR-TB
POR3	2007	4.3.4.2	LAM1	R	R	R	**R**	R	R	**R**	**R**	**R**	R	R	S	S	**R**	XDR-TB
POR4	2007	4.3.4.2	LAM1	R	R	R	R	R	R	R	**R**	S	R	R	S	S	**R**	XDR-TB
POR5	2007	4.3.4.2	LAM4	R	R	**R**	R	R	R	R	S	S	S	S	S	S	-	MDR-TB
POR6	2008	4.3.4.2	LAM4	R	R	**R**	R	R	R	R	R	R	R	R	S	S	R	XDR-TB
POR7	2009	4.3.4.2	LAM4	R	R	R	R	R	R	R	R	R	R	R	S	S	**R**	XDR-TB
POR8	2012	4.3.4.2	LAM4	R	R	**R**	R	R	R	R	R	R	R	R	S	S	R	XDR-TB
POR9	2011	4.3.4.2	LAM4	R	R	R	**R**	R	R	**R**	**R**	**R**	R	R	R	S	**R**	XDR-TB
POR10	2013	4.2.1	Ural H3/4	R	R	R	R	R	R	R	S	S	S	S	S	S	**R**	MDR-TB
H37Rv	-	4.9	H37RV	S	S	S	S	S	S	S	S	S	S	S	S	S	-	Pan-susceptible

*MDR-TB* multidrug-resistant TB, *XDR-TB* extensively drug-resistant TB, *INH* isoniazid, *RIF* rifampicin, *STR* streptomycin, *ETB* ethambutol, *PZA* pyrazinamide, *RFB* rifabutin, *ETH* ethionamide, *AMK* amikacin, *CAP* capreomycin, *OFX* ofloxacin, *MOX* moxifloxacin, *PAS* para-aminosalicylic acid, *LZ* linezolid, *KAN* kanamycin, *S* “susceptible”, *R* “resistant”

Bold indicates discrepant calls by *Mykrobe Predictor*, underlining indicates discrepant calls by *TBProfiler*

^a^Year of collection

^b^Drug susceptibility test not performed, with status inferred by the *TBProfiler* library

DNA was extracted and purified from the liquid cultures using a cetyltrimethylammonium bromide (CTAB) method [14]. The quality was assessed by fluorometric quantification, Qubit™ 3.0 Fluorometer with a dsDNA Broad Range Assay Kit (Thermo Fisher Scientific) and agarose gel electrophoresis. Triplicate DNA samples from each clinical isolate were prepared (biological replicates) and individual DNA extracts were subjected to repeated sequencing (technical replicates).

### Library preparation and sequencing

For MiSeq sequencing, ~200 ng of genomic DNA was sheared to an average size of 500 bp by ultrasonication (Covaris S220). Sheared DNA was purified/concentrated on MinElute Spin Columns (Qiagen). DNA concentrations were measured on a Nanodrop UV spectrophotometer and the sheared samples diluted to 5–12.5 ng/μl. Library constructions were performed using the Ovation Rapid DR Multiplex System (NuGen) according to the manufacturer’s instructions. Purified libraries were amplified in emulsion PCR, size selected (500–700 bp) by preparative electrophoresis on composite gels (1.2% LMP-Agarose/0.8% Synergel) and then purified on MinElute Columns. Libraries were sequenced with an Illumina MiSeq V3 and 300-bp paired-end reads with samples randomised across two runs (each ~24 h in duration).

Ion Torrent library preparation and sequencing was performed at Thermo Fisher Scientific, UK. Sequencing was carried out with the Ion Torrent PGM™ system (Ion PGM). Libraries were constructed with the Ion Xpress™ Plus Fragment Library Kit as per the manufacturer’s instructions (MAN0009847 Revision C.0), using 100 ng of genomic DNA which was sheared with the provided Ion Shear™ Plus Reagents to an average size of 350 bp, size selection using an E-Gel® SizeSelect™ 2% Agarose Gel, and purification with Agencourt® AMPure® XP Reagent. Finally, the libraries were quantified on the StepOnePlus™ System using the Ion Library Quantitation Kit, then diluted to 100 pM and pooled in equal volume. Purified libraries were sequenced with an Ion 318™ v2 chip (400-bp kit) and the Ion PGM™ HiQ™ Chef Kit as stated in the manual (MAN0010919, revision A.0). The runtime was ~3 h per sample. The software used on both Ion PGM™ and the Ion Chef™ System was Torrent Suite™ Software version 4.6.

### Bioinformatic pipeline

For the bioinformatic analysis we used a previously reported pipeline [10, 15, 16]. Unless stated otherwise, software was run at default settings. Reads were trimmed by *Trimmomatic* using a PHRED quality of 20 as the cutoff. Trimmed reads were then mapped against H37Rv (GCA_000195955.2) with *BWA-mem* v0.7.12 [17]. SNP and insertion and deletion (indel) variants were called with *Samtools* 0.1.19 [18] and *GATK* v3.6 [19]. We compared the variants called by both algorithms, but also report results of the conservative and typical approach of retaining the consensus polymorphisms across both methods. The genotypes of SNPs were called when an alternative allele was found in 20% of the mapped reads at a particular position. A default minimum depth of ten reads was required to call SNP genotypes, otherwise genotypes were denoted as missing data. This cutoff has been applied widely [15, 16, 20]. The robustness of the genotype calls was assessed across a range of depths of coverage of the reference and alternative alleles (depth 5–20, major allelic frequency >0.5 and >0.7). The reference genome was partitioned into overlapping 300-bp sequences allowing the uniqueness of genomic regions to be determined using *gem-mappability* [21]. Only 1.5% of the genome was estimated to be non-unique, and variants within these regions were discarded, leaving a set of high quality SNPs and indels. All 36 candidate drug-resistance genes [5] were found to be unique, thus removing the risk of false calling of SNPs due to inappropriate mapping to an analogous region. A summary of the pipeline is presented in Additional file 1: Figure S1.

### In silico profiling of *M. tuberculosis* resistance phenotypes

We compared two informatics tools for assigning resistance from sequence data. Drug-resistance status across 14 drugs was called in silico from raw sequence data using the web-based *TBProfiler* tool (http://tbdr.lshtm.ac.uk/) [5]). This tool also generates lists of mutations in candidate loci, and these formed the basis of identifying any additional putative novel polymorphisms. All mutations were checked by analysis of alignments and de novo assembly, as well as confirmed by alternative sequencing methods (see the next section, “Confirmation of mutations detected by whole genome sequencing”). Resistance profiles were also generated with the *Mykrobe predictor* tool (version July 2016) [22].

### Confirmation of mutations detected by whole genome sequencing

Genomic DNA was extracted as described above and used for PCR amplification prior to examination by line probe assay and/or DNA sequencing. The Genotype MTBDR*plus* (Hain Lifescience) investigates the *rpoB* and *katG* genes and *inhA* regulatory region and Genotype MTBDR*sl* (version 1, Hain Lifescience) investigates *rrs*, *gyrA* and *embB*. Both kits were used according to the manufacturer’s instructions. As the line probe assays encompass a limited number of loci, we also performed Sanger sequencing for *inhA*, *katG*, *tlyA*, *eis*, *gidB*, *pncA*, *gyrA*, *ethA*, *embB*, *embC-embA*, *rpsL*, *folC* and *thyX* genes (see Additional file 2: Table S1 for the primers used). PCR products were purified and both strands sequenced at StabVida (Portugal). All sequences were edited and analysed with ChromasPro 2.0.0 (Technelysium, Australia), compared to the sequences of *M. tuberculosis* H37Rv reference strain (GenBank AL123456.2) and aligned with Clustal Omega [23].

## Results

### Coverage

Triplicate “extraction” DNA samples from ten clinical isolates and a single H37Rv sample were sequenced on the MiSeq platform. Four DNA samples (from POR5, 6 and 7 and H37Rv) were each sequenced six times (“technical” replicates). Duplicate DNA samples from three clinical isolates (POR1, 2 and 6) were also sequenced on the Ion PGM. Summaries of the sequence data obtained for each platform are presented in Additional file 3: Table S2. With MiSeq sequencing the number of paired reads varied across samples (median 1.2 million, range 0.4 to 3.2 million), and on average 99% of reads mapped to the H37Rv reference, giving a median depth of coverage of 51-fold (across sample range 18- to 79-fold). The majority of the genome (>96%) was covered to at least tenfold depth.

**Fig. 1:**
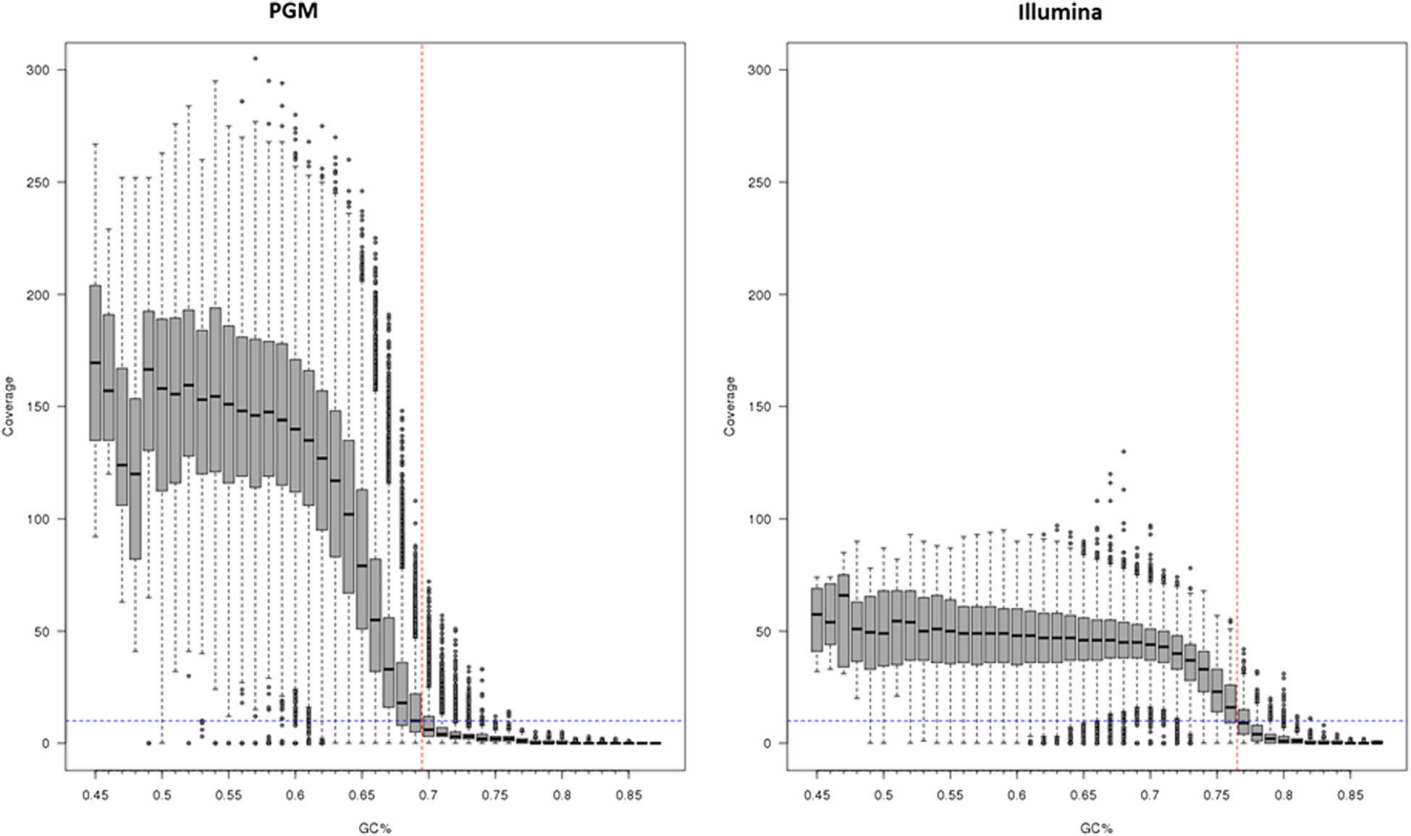
The dependence of coverage on GC content. The coverage across regions of the genome with differing GC content compared using two different sequencing technologies; the Ion PGM and the Illumina MiSeq. The *dashed blue line* represents the cutoff used when calling variants. Any position which had a coverage <10 was marked as missing. The *dashed red line* shows at which GC% the median coverage across the window falls below the cutoff

**Fig. 2:**
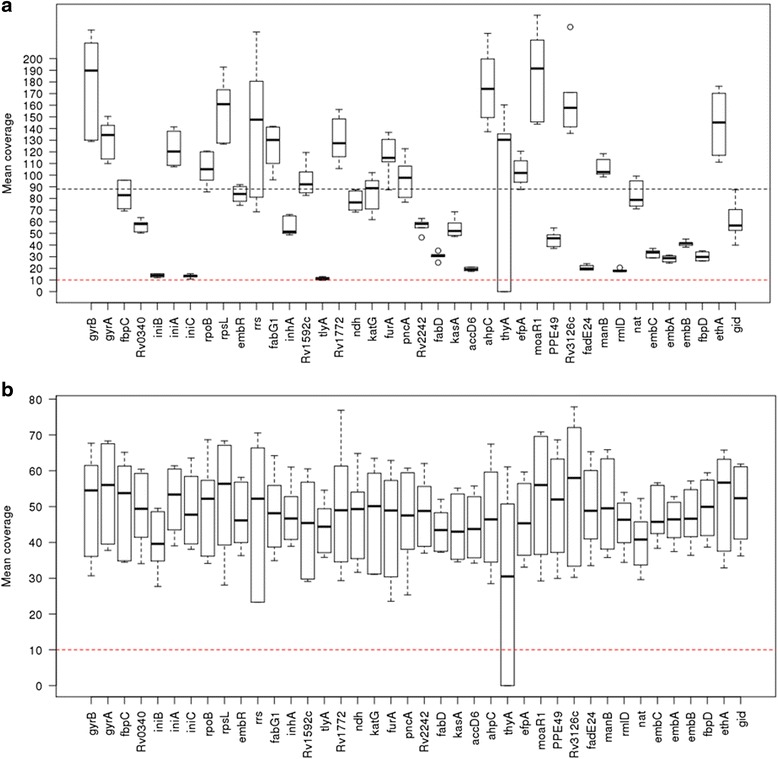
Coverage across drug-resistance genes. The coverage across the drug-resistance genes in POR1, 2 and 6 samples sequenced using both the **a** Ion PGM and **b** Illumina MiSeq. The *dashed red line* represents the cutoff used when calling variants. Any position with less than tenfold coverage was marked as missing. The low coverage in *thyA* is due to a deletion polymorphism

**Fig. 3:**
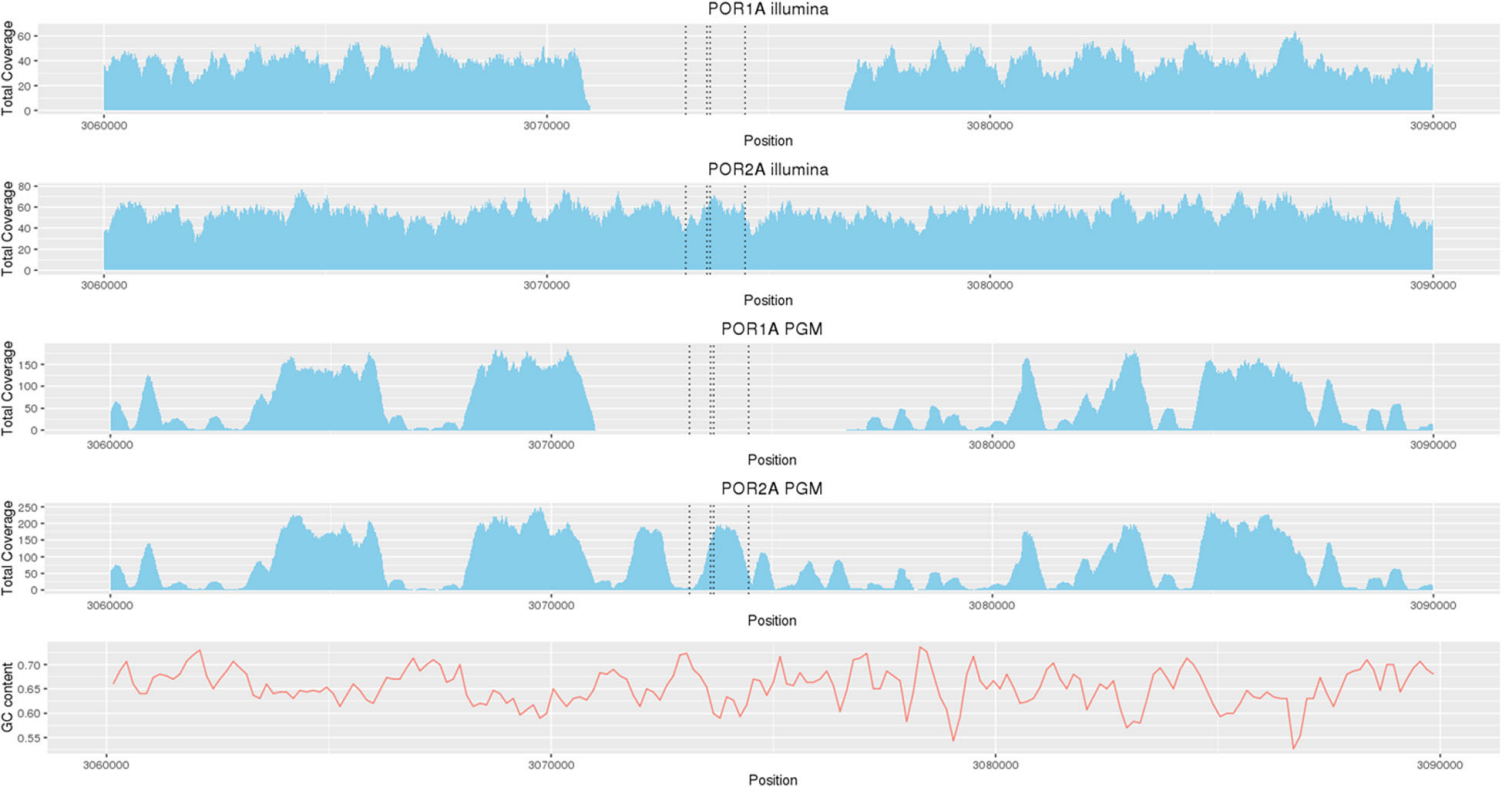
Lack of genomic coverage in *dfrA-thyA* genes reveals deletions in the POR1A XDR isolate with PAS resistance. Uneven Ion PGM sequence coverage is due to high GC content

In contrast, for the Ion PGM platform the median number of reads was 990,854 (range 928,006–1,124,215) translating into a median of 53-fold (range 48- to 59-fold) genomic coverage. A large proportion of the genome (~25%) had low coverage and was attributed to regions with high GC content (Fig. 1). Whilst high coverage (100- to 200-fold) was attained for regions with GC content up to 69%, above this level coverage drops below tenfold, which was the cutoff used for calling variants. For MiSeq sequence data, this drop only occurs when the GC content reaches 75% or above. Many regions in the *M. tuberculosis* genome, especially the *pe/ppe* genes [24], are high in GC content (median 69%, range 47–87%) and therefore potentially difficult to characterise. The coverage across the 36 drug-resistance candidate genes was high for MiSeq (mean ~90-fold) and exceeded the tenfold cutoff, except in the *thyA* gene in the three POR1 replicates (Fig. 2). These XDR-TB replicates contained double *dfrA*-*thyA* deletions, thought to be responsible for para-aminosalicylic acid (PAS) resistance [25]. A direct comparison of the POR1, 2 and 6 sample coverage across platforms highlighted greater variability in candidate genes in Ion PGM due to differential GC content. Whilst there was platform-wide detection of the deletion-driven lower coverage in *thyA* in POR1 (Fig. 3; Additional file 4: Figure S2), the variable coverage in the neighbouring regions for Ion PGM could lead to less certainty in detection.

### SNP variants and error rates

We estimated the variant error rates (measured as the number of sites which were discordant among replicates) to be low for both platforms (MiSeq 1 per 77 kbp, Ion PGM 1 per 41 kbp). Across comparable samples, the number of high quality SNPs detected using MiSeq data was higher than from Ion PGM, mostly due to low coverage in the alignments generated from the Ion PGM (Additional file 3: Table S2). We sought to investigate the effects of variant calling algorithms on the numbers of SNPs detected in unique genomic regions. From the MiSeq H37Rv data, similar numbers of SNPs were detected across replicates (*Samtools* 64–69 SNPs and *GATK* 69–79 SNPs, overlap 69 SNPs), supporting the existence of those variants and high sequence reproducibility (Additional file 5: Table S3). Across clinical isolate replicates the number of SNPs identified was similar and the overlap between variant calling algorithms was high (>90%; Additional file 5: Table S3). This observation was supported by the Ion PGM data but, due to uneven coverage, at least 120 SNPs fewer were identified when compared to matching MiSeq samples. Within platforms and calling algorithms there was variation between replicates in the indels detected, but there was high overlap between algorithms (>90%; Additional file 5: Table S3). Compared to SNPs the breakpoints for these variants are more difficult to characterise from alignments.

For the MiSeq platform data we assessed the number of SNP genotypes called across a range of coverage depths of the reference and alternative alleles (total depth 5- to 20-fold; major allelic frequency >0.5 and >0.7). The number of SNPs decreased pseudo-linearly with decreasing minimum read depth for H37Rv (87 to 67 SNPs; Additional file 6: Figure S3) and the ten clinical isolates (2290 to 2097 SNPs; Additional file 7: Figure S4). In general, differences in the number of SNPs between the *Samtools* and *GATK* algorithms decreased as the depth of coverage and allelic frequency thresholds increased. For H37Rv, read depths in excess of 20-fold had no impact on variants detected. Across clinical isolates, the highest possible stringency tested consisted of using a minimum coverage of 20 and an allelic frequency of 0.7 and led to near identical numbers of total SNPs called by the two variant calling algorithms (*Samtools* 1943, *GATK* 1990, either 2097, both 1840 SNPs; Additional file 7: Figure S4). Much of the discordance in the number of SNPs within replicate groups is due to differences in coverage leading to some polymorphisms not passing quality control filters. Using SNPs for which all replicates have non-missing genotypes, all replicates had identical numbers of SNPs except POR3C, which differed by two SNPs between POR3A and POR3B. Overall, the analyses indicated no major differences in SNPs detected between the two calling algorithms, and this supported the use of consensus variants for downstream analysis. For example, the set of common SNP variants were used to cluster all samples within a phylogenetic tree using *FastTree* v2.1.7 [26] (Additional file 8: Figure S5). Perfect clustering was observed amongst isolates and their replicates. At a finer resolution, we analysed the SNP differences between the replicates, and none were identified.

### Calling in silico resistance phenotypes

When the MiSeq raw sequence data were subjected to analysis using *TBProfiler*, agreement with phenotypic susceptibility testing was high (95.3%, 82/86; Table 1). Discrepant results were recorded for PZA (×2) and PAS (×2) where phenotypically resistant isolates not identified by *TBProfiler* were found to have novel mutations in known candidate genes (Additional file 9: Table S4). The novel polymorphisms included a deletion in *pncA* of 20 bp (nucleotides 437–449) and a nucleotide insertion (GG) between codons 130 and 131. PAS-resistant isolates had a *folC* S98G mutation and a *thyX G*-4A, *thyX I161T*, *dfrA-thyA* deletion. Phenotypic testing of kanamycin drug susceptibility was not performed, but mutations associated with its resistance were detected in all eight isolates (Table 1; Additional file 9: Table S4). All mutations were confirmed using independent Sanger capillary sequencing and/or the line probe assays Genotype MTBDR*plus* and Genotype MTBDR*sl* (Hain). Phenotypic resistance profiles were confirmed and quantified by the qDST method for the MGIT960 system [12, 13].

The *Mykrobe predictor* tool was also applied to in silico call resistance. This approach looks for mutations associated with resistance to first-line drugs (rifampicin, isoniazid, ethambutol) and second-line drugs (streptomycin, ciprofloxacin, ofloxacin, moxifloxacin, amikacin, kanamycin, capreomycin). Of the 72 resistance calls made, 19 (26.4%) were incorrectly called “susceptible”. False negative calls were made for isoniazid (×1), ethambutol (×2), streptomycin (×4), amikacin (×4), and capreomycin (×3). Additionally there was a disagreement between *TBprofiler* and *Mykrobe predictor* with four samples for kanamycin, the latter program calling them as “susceptible” (Table 1).

For Ion PGM, when predicting individual drug-resistance profiles in the three isolates, in one isolate the *gyrA* D94A mutation associated with fluoroquinolone resistance could not be detected due to lack of coverage (Additional file 5: Table S3). However, the mutation was recovered if the coverage threshold was relaxed from ten- to fourfold.

## Discussion

Advances in next-generation sequencing technology have expanded opportunities for genome analysis in the clinical laboratory. Determining resistance to anti-TB drugs by whole genome sequencing has been demonstrated as feasible and is being implemented in some specialist centres [6]. For acceptance as a diagnostic tool to guide treatment of drug-resistant TB the sequencing platforms and analytical tools employed must be robust and reliable. Here we have investigated the performance of two commercial ‘bench-top’ next generation sequencing platforms and attempted to assess the robustness of a bioinformatics analysis pipeline with respect to variant calling, across sequencing replicates.

The MiSeq and Ion PGM both proved satisfactory for determining drug-resistance profiles. Compared to Ion PGM, MiSeq sequence coverage was more uniform and was better represented in regions with high GC content. However, we did not investigate the impact of the different library preparation methods used (mechanical (MiSeq) and enzymatic (Ion PGM) processing). Sample quality and the mode or preparation have been shown to influence the depth of coverage in high GC regions [27], and further work is required to investigate this. The Ion PGM platform has previously been used to characterise mutations in XDR-TB strains [6], but the minimum read depth used to call alleles (fourfold) were less stringent than the tenfold coverage threshold adopted here.

*Samtools* and *GATK* when used to process the raw sequence data produced diverse outputs but filtering based on coverage and allelic frequency led to almost complete agreement on resistance causing SNPs. There was, however, lower concordance between the final sets of indels. As previously reported, the false discovery rate for *Samtools* is higher than for *GATK* and rises as coverage increases [28]. A common strategy is to undertake dual analysis and consider the intersection of the *Samtools* and *GATK* derived SNPs but select only the *GATK* indels [16]. The high reproducibility of sequence data from replicate samples is reassuring as it affirms the validity of next-generation sequencing as a tool for investigating transmission events.

Of the two rapid tools examined, the *TBProfiler* gave 100% concordance with phenotypic DST results for INH, RIF, STR, ETB, ETH and the fluoroquinolones. Of the nine PZA-resistant isolates, known resistance SNPs were reported for seven isolates with an insertion and deletion observed for the remaining two. Possible novel resistance mutations were also observed for both the PAS-resistant isolates. The *Mykrobe predictor* detected resistance for nine drugs, of which eight had DST results. Concordance was 100% for RIF, OFX and MOX, but resistance was missed for one or more isolates for the remaining five drugs. Misclassification of resistance of amikacin and capreomycin as susceptible has significant clinical implications as patients may be assigned treatment that is not effective for XDR-TB.

The identification of a PAS resistance-related *dfrA-thyA* double deletion in an XDR-TB sample highlights the need to look at non-SNP variants. Significantly, the laboratory platform being used may impact the detection of putative drug resistance. This is critical in XDR-TB and resistance beyond XDR-TB where use of drugs like PAS may make the difference in providing a life-saving effective regimen of at least five drugs [29]. Large deletions and other structural variants may be detected by applying a combination of complementary approaches (pair-end, split-read and depth of coverage) followed by a validation process involving de novo assembly of bordering reads and re-alignment to the reference genome [10, 16, 24]. However, high genome-wide sequence coverage is necessary to perform such analyses.

As expected the genotypic profiling was concordant with the phenotypic determination of drug-resistance levels confirming the reliability and robustness of the selected genes and mutations as predictors of resistance for almost all drugs tested; with discrepancies still being noticed for PZA and PAS due to lack of enough information on their mechanism of action [12, 30]. Surprisingly, no discrepancies were found for EMB, a drug known to have low correlation between the *emb* genes and phenotypic resistance [12].

## Conclusions

Sequencing platforms are becoming more accessible and economical. Our work suggests that they are capable of delivering high quality data regarding resistance to anti-TB drugs but do not all perform to the same standard and quality monitoring is advisable. Further studies are needed to evaluate these analytical tools, which as yet do not have regulatory approval for clinical use. It is expected that drug-resistance profiling using next-generation sequencing will gain accuracy and reliability with the gathering of improved knowledge of the drug-target genes and resistance-causing mutations, including for the new drugs recently approved for the treatment of MDR- and XDR-TB [29, 31]. Ultimately, drug resistance profiling using next-generation sequencing offers rapid assessment of resistance-associated mutations, thus accelerating access to effective treatment.

## Additional files

Additional file 1: Figure S1. Bioinformatics pipeline. (TIFF 81 kb)
Additional file 2: Table S1. Sanger sequencing primers for genomic variant confirmation. (DOCX 132 kb)
Additional file 3: Table S2. Summary of the sequencing data, coverage and SNPs for each sample. (DOCX 22 kb)
Additional file 4: Figure S2.
**a** Mean coverage for all samples for each drug resistance gene. Deletion of *dfrA-thyA* is evident by the zero coverage outliers in POR1. **b** Mean coverage across drug-resistance genes. (TIFF 273 kb)
Additional file 5: Table S3. Replicate variation across extraction and calling algorithms, and phenotypic profiles. (DOCX 20 kb)
Additional file 6: Figure S3. The changes in the number of SNPs characterised across algorithms for H37Rv. (TIFF 85 kb)
Additional file 7: Figure S4. The changes in the number of SNPs characterised across algorithms for the ten clinical isolates. (TIFF 92 kb)
Additional file 8: Figure S5. Phylogenetic tree of all the MiSeq sequenced samples. (TIFF 76 kb)
Additional file 9: Table S4. Mutations that potentially explain drug resistance in the samples. (DOCX 19 kb)
