# Supplementary figures and images for: The variability and reproducibility of whole genome sequencing technology for detecting resistance to anti-tuberculous drugs
Source: Genome Med. 2016 Dec 22;8:132. doi: 10.1186/s13073-016-0385-x (PMC5178084; doi:10.1186/s13073-016-0385-x)

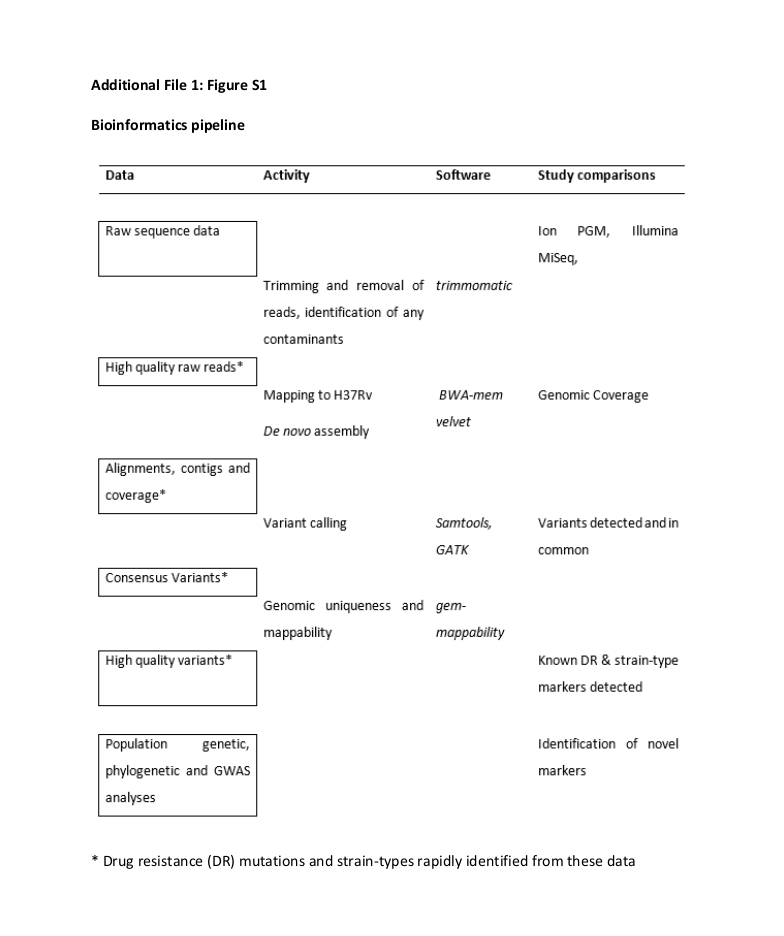

Supplement: Additional file 1: Figure S1. — Bioinformatics pipeline. (TIFF 81 kb) [file 13073_2016_385_MOESM1_ESM.tiff]

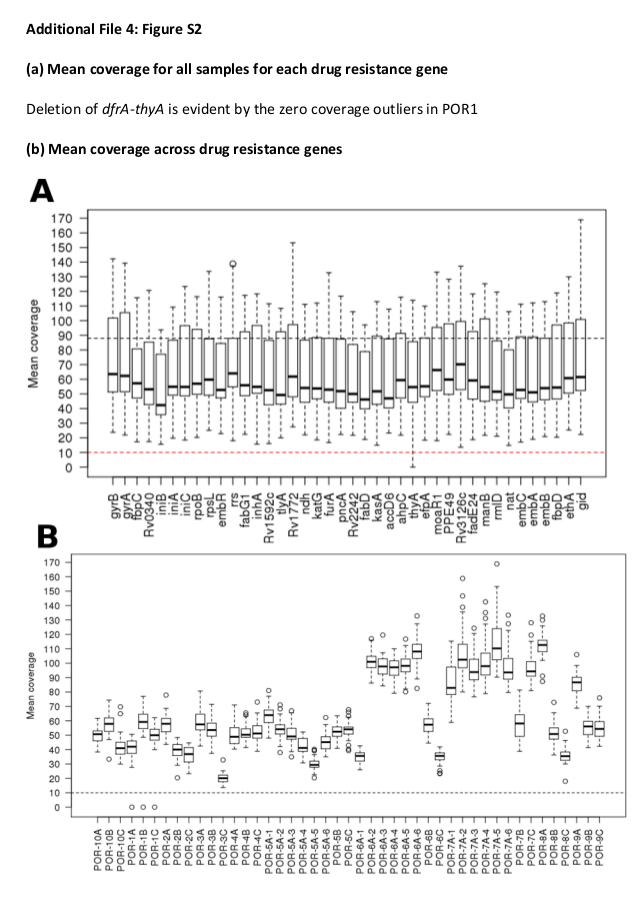

Supplement: Additional file 4: Figure S2. — a Mean coverage for all samples for each drug resistance gene. Deletion of dfrA-thyA is evident by the zero coverage outliers in POR1. b Mean coverage across drug-resistance genes. (TIFF 273 kb) [file 13073_2016_385_MOESM4_ESM.tiff]

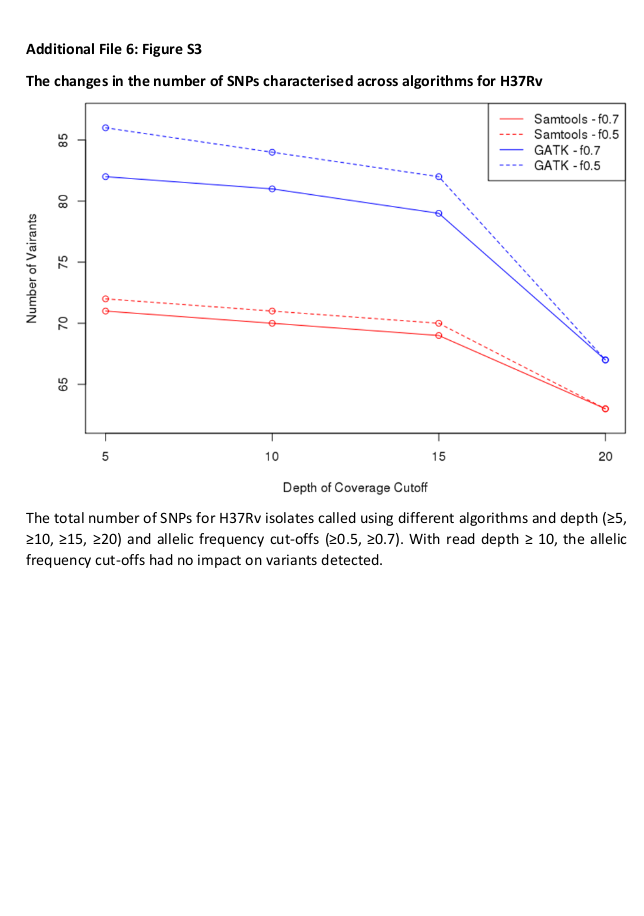

Supplement: Additional file 6: Figure S3. — The changes in the number of SNPs characterised across algorithms for H37Rv. (TIFF 85 kb) [file 13073_2016_385_MOESM6_ESM.tiff]

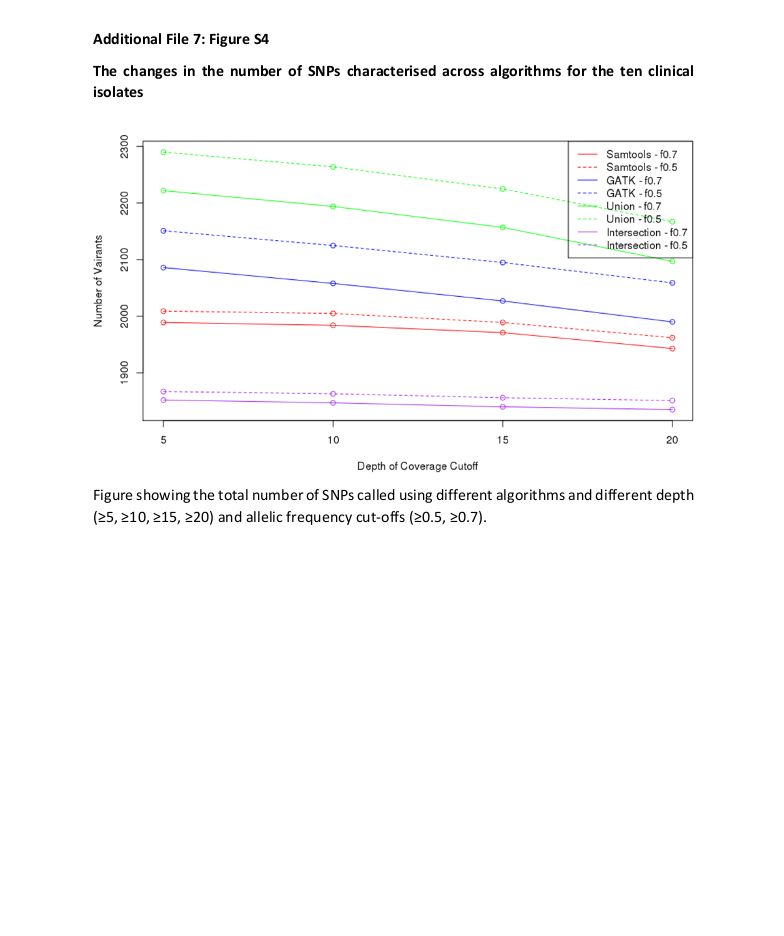

Supplement: Additional file 7: Figure S4. — The changes in the number of SNPs characterised across algorithms for the ten clinical isolates. (TIFF 92 kb) [file 13073_2016_385_MOESM7_ESM.tiff]

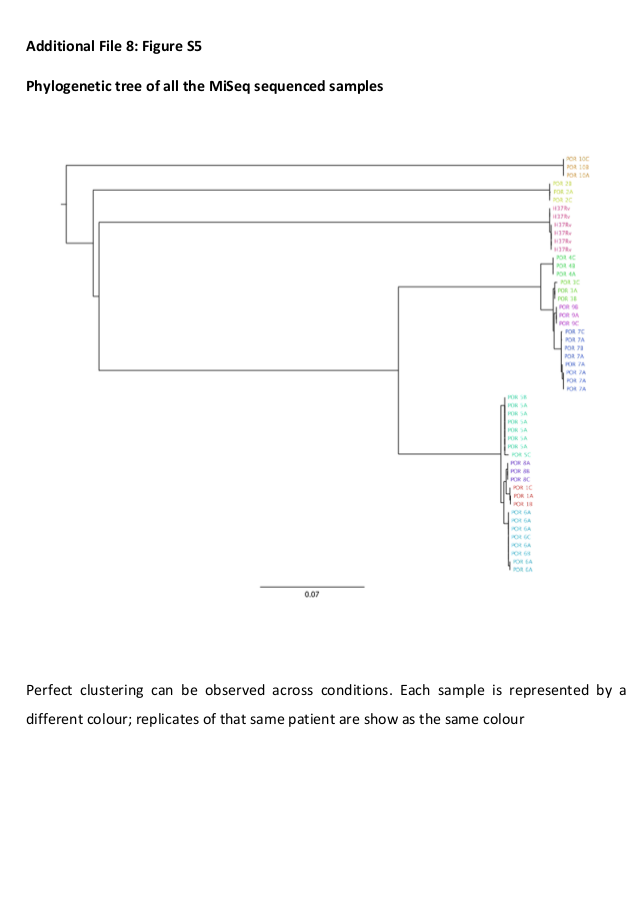

Supplement: Additional file 8: Figure S5. — Phylogenetic tree of all the MiSeq sequenced samples. (TIFF 76 kb) [file 13073_2016_385_MOESM8_ESM.tiff]
