# Supplementary material for: The variability and reproducibility of whole genome sequencing technology for detecting resistance to anti-tuberculous drugs
Source: Genome Med. 2016 Dec 22;8:132. doi: 10.1186/s13073-016-0385-x (PMC5178084; doi:10.1186/s13073-016-0385-x)
Supplement: Additional file 2: Table S1. — Sanger sequencing primers for genomic variant confirmation. (DOCX 132 kb) [file 13073_2016_385_MOESM2_ESM.docx]

**Additional File 2: Table S1**

**Sanger sequencing primers for genomic variant confirmation**

| **Gene** | **Primer** | **Primer sequence (5’-3’)** | **Annealing (^0^C)** | **Length (bp)** | **Ref.** |
| --- | --- | --- | --- | --- | --- |
| ***inhA*** | *inhA-1* | CCT CGC TGC CCA GAA AGG GA | 64 | 248 | A |
|  | *inhA-2* | ATC CCC CGG TTT CCT CCG GT |  |  |  |
|  | *inhA-3* | AGG TCG CCG GGG TGG TCA GC | 60 | 517 |  |
|  | *inhA-4* | AGC GCC TTG GCC ATC GAA GCA |  |  |  |
|  | *inhA-3F* | CCA CAT CTC GGC GTA TTC G |  | 501 | B |
|  | *inhA-5R* | TTC CGG TCC GCC GAA CGA CAG |  |  |  |
| ***katG*** | *P4_Fw* | CGG ACC ATA ACG GCT TCC TG | 62 | 563 | C |
|  | *P4_Rv* | TTG TCC AAG CTG GCG TTG TC |  |  |  |
|  | *P5_Fw* | CGA CAA CGC CAG CTT GGA C |  | 518 |  |
|  | *P5_Rv* | CGG TTC CGG TGC CAT ACG |  |  |  |
|  | *P6_Fw* | AGC TCG TAT GGC ACC GGA AC |  | 619 |  |
|  | *P6_Rv* | TGA CCT CCC ACC CGA CTT GT |  |  |  |
|  | *P7_Fw* | ACA AGT CGG GTG GGA GGT C |  | 574 |  |
|  | *P7_Rv* | CTG CCG GTC CAC TTC ACC TT |  |  |  |
|  | *P8_Fw* | GGG ACC TAC CAG GGC AAG GA |  | 629 |  |
|  | *P8_Rv* | CCG GGA GTC AGC AAG TCA CC |  |  |  |
| ***tlyA*** | *tlyAs* | GCA TCG CAC GTC GTC TTT | 55 | 947 | D |
|  | *tlyAas* | GGT CTC GGT GGC TTC GTC |  |  |  |
| ***eis*** | *eisF1* | GCC ATG GGA CCG GTA CTT GC | 56 | 601 | E |
|  | *eisR1* | GTA GAT GCC GCC CTC GCT AG |  |  |  |
| ***gidB*** | *gidB_Fw* | CGA GAG CGG AGA ATG TTT CA | 62 | 793 | F |
|  | *gidB_Rv* | CTG GCC CGA CCT TAC GAG |  |  |  |
| ***pncA*** | *pncA_promP1* | GCT GGT CAT GTT CGC GAT CG | 55 | 214 | G |
|  | *pncA_promP2* | TCG GCC AGG TAG TCG CTG AT |  |  |  |
|  | *pncA_Fw* | AGT CGC CCG AAC GTA TGG TG | 62 | 615 | H |
|  | *pncA_Rv* | CAA CAG TTC ATC CCG GTT CG |  |  |  |
| ***gyrA*** | *gyrA_Fw* | ATC GCC GGG TGC TCT ATG | 62 | 321 | F |
|  | *gyrA_Rv* | GGC CGT CGT AGT TAG GGA TG |  |  |  |
| ***ethA*** | *ethA1* | ATC ATC GTC GTC TGA CTA TGG | 55 | 667 | A |
|  | *ethA5* | ACT ACA ACC CCT GGG ACC |  |  |  |
|  | *ethA4* | CCT CGA CCT TCC CGT GA | 64 | 692 |  |
|  | *ethA9* | CCT CGA GTA CGT CAA GAG CAC |  |  |  |
|  | *ethA8* | GGT GGA ACC GGA TAT GCC TG | 68 | 342 |  |
|  | *ethA10* | CGT TGA CGG CCT CGA CAT TAC |  |  |  |
| ***embB*** | *embB-F2* | AAC CTG CGC CCG CAG ATT GTC | 62 | 526 | I |
|  | *embB-R2* | GGT CTG GCA GGC GCA TCC |  |  |  |
|  | *embBR2_Fw* | CTG GCG CTG ATG ACC CAT | 62 | 588 | * |
|  | *embBR2_Rv* | GGT GGG CAG GAT GAG GTA G |  |  |  |
| ***embC-embA* IRG** | *embC-embA_Fw* | GGT TGA CGC CTT ACT ACC C | 62 | 535 | J |
|  | embC-embA_Rv | CCA CGA CGA CCG TGT CC |  |  |  |
| ***rpsL*** | *rpsL_Fw* | GGC CGA CAA ACA GAA CGT | 64 | 504 | K |
|  | *rpsL_Rv* | GTT CAC CAA CTG GGT GAC |  |  |  |
| ***folC*** | *folCP1*-Fw | CGC TGC AAT GAA TTC GAC GA | 62 | 668 | * |
|  | *folCP1*-Rv | TGA TGA TGC CCG CCT TCT C |  |  |  |
| ***thyX*** | *thyX*prom_Fw | TGG ATG GAA AAC CTT GCG G | 62 | 558 | * |
|  | *thyX*prom_Rv | TCG GTC TTG GCG ATC AGT T |  |  |  |
|  | *thyX*-F2 | CTA CTC GCA GCT CTC CCA G | 62 | 510 | * |
|  | *thyX*-R2 | TAC CTG GCG CTT TAT CCC G |  |  |  |

[A] Morlock G, Metchock B, Sikes D, Crawford J, Cooksey R. ethA, inhA, and katG loci of ethionamide-resistant clinical Mycobacterium tuberculosis isolates. Antimicrob Agents Chemother 2003; 47: 3799-805.

[B] Leung E, Ho P, Yuen K, Woo W, Lam T, Kao R, Seto W, Yam W. Molecular characterization of isoniazid resistance in Mycobacterium tuberculosis: identification of a novel mutation in inhA. Antimicrob Agents Chemother 2006; 50: 1075-8.

[C] Machado D, Perdigão J, Ramos J, Couto I, Portugal I, Ritter C, Boettger E, Viveiros M. High-level resistance to isoniazid and ethionamide in multidrug-resistant Mycobacterium tuberculosis of the Lisboa family is associated with inhA double mutations. J Antimicrob Chemother. 2013; 68: 1728-32.

[D] Feuerriegel S, Cox H, Zarkua N, Karimovich H, Braker K, Rüsch-Gerdes S, Niemann S. Sequence analyses of just four genes to detect extensively drug-resistant Mycobacterium tuberculosis strains in multidrug-resistant tuberculosis patients undergoing treatment. Antimicrob Agents Chemother. 2009; 53: 3353-6.

[E] Perdigão J, Macedo R, Silva C, Machado D, Couto I, Viveiros M, Jordão L, Portugal I. From multidrug-resistant to extensively drug-resistant tuberculosis in Lisbon, Portugal: the stepwise mode of resistance acquisition. J Antimicrob Chemother. 2013; 68: 27-33

[F] Machado, D. (2014). The dynamics of drug resistance in Mycobacterium tuberculosis: exploring the biological basis of multi- and extensively drug resistant tuberculosis (MDR/XDRTB) as a route for alternative therapeutic strategies. PhD thesis. Instituto de Higiene e Medicina Tropical, Universidade Nova de Lisboa. Lisboa, Portugal.

[G] Scorpio A, Lindholm-Levy P, Heifets L, Gilman R, Siddiqi S, Cynamom M, Zhang Y. Characterization of pncA mutations in pirazinamide-resistant Mycobacterium tuberculosis. Antimicrob Agents Chemother. 1997; 41: 540-3.

[H] Louw G, Warren R, Donald P, Murray M, Bosman M, Van Helden P, Young D, Victor T. Frequency and implications of pyrazinamide resistance in managing previously treated tuberculosis patients. Int J Tuberc Lung Dis. 2006; 10: 802-7.

[I] Starks A, Gumusboga A. Plikaytis B, Shinnick T, Posey J. Mutations at embB306 are an important molecular indicator of ethambutol resistance in Mycobacterium tuberculosis. Antimicrob Agents Chemother. 2009; 53: 1061-66.

[J] Cui Z, Li Y, Cheng S, Yang H, Lu Junmei, Hu Z, Ge B. Mutations in the embC-embA intergenic region contribute to Mycobacterium tuberculosis resistance to ethambutol. Antimicrob Agents Chemother. 2014; 58: 6837-43.

[K] Sreevatsan S, Pan X, Stockbauer K, Williams D, Kreiswirth B, Musser J. Characterization of rpsL and rrs mutations in streptomycin-resistant Mycobacterium tuberculosis isolates from diverse geographic localities. Antimicrob Agents Chemother. 1996; 40: 1024-6.

* This work
