# Supplementary material for: The variability and reproducibility of whole genome sequencing technology for detecting resistance to anti-tuberculous drugs
Source: Genome Med. 2016 Dec 22;8:132. doi: 10.1186/s13073-016-0385-x (PMC5178084; doi:10.1186/s13073-016-0385-x)
Supplement: Additional file 3: Table S2. — Summary of the sequencing data, coverage and SNPs for each sample. (DOCX 22 kb) [file 13073_2016_385_MOESM3_ESM.docx]

**Additional File 3: Table S2**

**Summary of the sequencing data, coverage and SNPs for each sample**

| Sequencing platform | Sample | No. reads | Median read length | Proportion coverage | Median coverage | Total SNPs |
| --- | --- | --- | --- | --- | --- | --- |
|  |  |  |  | > 10-fold |  |  |
| MiSeq | POR1A | 874721 | 222 | 0.95 | 40 | 766 |
| MiSeq | POR1B | 1280618 | 221 | 0.96 | 55 | 766 |
| MiSeq | POR1C | 1068336 | 221 | 0.96 | 48 | 766 |
| Ion PGM | POR1A | 1015193 | 335 | 0.73 | 48 | 512 |
| Ion PGM | POR1B | 1124215 | 339 | 0.67 | 52 | 512 |
| MiSeq | POR2A | 1167341 | 224 | 0.97 | 53 | 854 |
| MiSeq | POR2B | 871084 | 223 | 0.97 | 38 | 854 |
| MiSeq | POR2C | 817606 | 224 | 0.97 | 36 | 854 |
| Ion PGM | POR2A | 929733 | 213 | 0.73 | 28 | 594 |
| Ion PGM | POR2C | 966514 | 326 | 0.74 | 46 | 594 |
| MiSeq | POR3A | 1217694 | 224 | 0.96 | 55 | 771 |
| MiSeq | POR3B | 1100251 | 222 | 0.96 | 50 | 771 |
| MiSeq | POR3C | 413660 | 215 | 0.93 | 18 | 773 |
| MiSeq | POR4A | 1055194 | 218 | 0.96 | 47 | 795 |
| MiSeq | POR4B | 1100448 | 224 | 0.96 | 50 | 795 |
| MiSeq | POR4C | 1071269 | 225 | 0.96 | 49 | 795 |
| MiSeq | POR5A* | 988848 | 224 | 0.96 | 45 | 758 |
| MiSeq | POR5B | 1111052 | 224 | 0.96 | 51 | 758 |
| MiSeq | POR5C | 1113854 | 223 | 0.96 | 50 | 758 |
| MiSeq | POR6A* | 2269310 | 180 | 0.97 | 70 | 767 |
| MiSeq | POR6B | 1201932 | 222 | 0.96 | 53 | 767 |
| MiSeq | POR6C | 774063 | 222 | 0.96 | 34 | 767 |
| Ion PGM | POR6B | 1049314 | 338 | 0.72 | 44 | 510 |
| Ion PGM | POR6C | 904304 | 325 | 0.73 | 42 | 510 |
| MiSeq | POR7A* | 2423026 | 179 | 0.97 | 70 | 801 |
| MiSeq | POR7B | 1129806 | 222 | 0.96 | 51 | 801 |
| MiSeq | POR7C | 2638858 | 155 | 0.97 | 65 | 801 |
| MiSeq | POR8A | 2851160 | 172 | 0.97 | 79 | 770 |
| MiSeq | POR8B | 1028634 | 225 | 0.96 | 49 | 770 |
| MiSeq | POR8C | 801687 | 222 | 0.96 | 36 | 770 |
| MiSeq | POR9A | 2091394 | 180 | 0.97 | 61 | 796 |
| MiSeq | POR9B | 1145983 | 225 | 0.96 | 53 | 796 |
| MiSeq | POR9C | 1128251 | 223 | 0.96 | 51 | 796 |
| MiSeq | POR10A | 1074170 | 217 | 0.97 | 48 | 902 |
| MiSeq | POR10B | 1224053 | 223 | 0.97 | 54 | 902 |
| MiSeq | POR10C | 894289 | 223 | 0.97 | 39 | 902 |
| MiSeq | H37Rv* | 2652971 | 156 | 0.99 | 56 | 62 |

* 6 technical replicates for each, and average statistics presented; A-C refers to extraction replicates of the same samples
