# Supplementary material for: The variability and reproducibility of whole genome sequencing technology for detecting resistance to anti-tuberculous drugs
Source: Genome Med. 2016 Dec 22;8:132. doi: 10.1186/s13073-016-0385-x (PMC5178084; doi:10.1186/s13073-016-0385-x)
Supplement: Additional file 5: Table S3. — Replicate variation across extraction and calling algorithms, and phenotypic profiles. (DOCX 20 kb) [file 13073_2016_385_MOESM5_ESM.docx]

**Additional File 5: Table S3**

**Replicate variation across extraction and calling algorithms, and phenotypic profiles**

| **Sequencing platform** | **Comparison**  **(no. rep, M/XDR-TB)** | **GATK**  **SNPs*** | ***Samtools***  **SNPs*** | ***Overlap***  ***SNPs %*** | ***GATK* Indels**  **Min. / total (overlap)** | ***Samtools* Indels**  **Min. / total (overlap)** | ***Overlap***  ***Indels %***** | **Inferred MDR/XDR-TB** | **Inferred drug resistance*****  **INH, RIF, ETH +** |
| --- | --- | --- | --- | --- | --- | --- | --- | --- | --- |
|  | *Technical* |  |  |  |  |  |  |  |  |
| MiSeq | POR5A (6,M) | 783 | 753 | 96.2 | 85/98 (0.87) | 66/104 (0.63) | 94.2 | MDR-TB | ETB, PZA, STR |
| MiSeq | POR6A (6,X) | 846 | 815 | 92.2 | 87/103 (0.84) | 65/127 (0.51) | 81.1 | XDR-TB | ETB, PZA, STR, FLQ, AMK, CAP, KAN |
| MiSeq | POR7A (6,X) | 858 | 839 | 97.1 | 90/102 (0.88) | 72/133 (0.54) | 76.7 | XDR-TB | ETB, PZA, STR, FLQ, AMK, CAP, KAN |
| MiSeq | H37Rv (6,S) | 81 | 70 | 84.1 | 22/27 (0.81) | 16/40 (0.40) | 67.5 | Susc. |  |
|  | *Extraction* |  |  |  |  |  |  |  |  |
| MiSeq | POR1 (3,X) | 788 | 753 | 95.6 | 86/91 (0.95) | 72/99 (0.73) | 91.9 | XDR-TB | ETB, PZA, STR, FLQ, AMK, CAP, KAN |
| Ion PGM | POR1 (2,X) | 618 | 611 | 95.7 | 48/98(0.49) | 53/81 (0.65) | 34.1 | XDR-TB | ETB, PZA, STR, FLQ, AMK, CAP, KAN |
| MiSeq | POR2 (3,M) | 875 | 846 | 96.7 | 99/114 (0.87) | 88/115 (0.77) | 99.1 | MDR-TB |  |
| Ion PGM | POR2 (2,M) | 710 | 706 | 96.3 | 23/52(0.44) | 39/67 (0.58) | 22.2 | MDR-TB |  |
| MiSeq | POR3 (3,X) | 804 | 788 | 97.8 | 87/98 (0.89) | 70/100 (0.70) | 98.0 | XDR-TB | ETB, STR, FLQ, AMK, CAP, KAN |
| MiSeq | POR4 (3,X) | 805 | 789 | 98.0 | 86/91 (0.95) | 69/92 (0.75) | 98.9 | XDR-TB | ETB, PZA, STR, FLQ, AMK, KAN |
| MiSeq | POR5 (3,M) | 784 | 754 | 96.2 | 90/92 (0.98) | 74/94 (0.79) | 97.9 | MDR-TB | ETB, PZA, STR |
| MiSeq | POR6 (3,X) | 849 | 827 | 90.9 | 87/98 (0.89) | 70/97 (0.72) | 99.0 | XDR-TB | ETB, PZA, STR, FLQ, AMK, CAP, KAN |
| Ion PGM | POR6 (2,X) | 617 | 612 | 95.7 | 33/76(0.43) | 46/82 (0.56) | 23.8 | **MDR-TB** | ETB, PZA, STR, AMK, CAP, KAN |
| MiSeq | POR7 (3,X) | 875 | 868 | 93.5 | 87/102 (0.85) | 73/104 (0.70) | 98.1 | XDR-TB | ETB, PZA, STR, FLQ, AMK, CAP, KAN |
| MiSeq | POR8 (3,X) | 820 | 791 | 94.1 | 84/96 (0.88) | 74/95 (0.78) | 99.0 | XDR-TB | ETB, PZA, STR, FLQ, AMK, CAP, KAN |
| MiSeq | POR9 (3,X) | 820 | 807 | 97.2 | 90/98 (0.92) | 77/104 (0.57) | 94.2 | XDR-TB | ETB, PZA, STR, FLQ, AMK, CAP, KAN |
| MiSeq | POR10 (3,M) | 922 | 885 | 95.8 | 98/108 (0.91) | 78/107 (0.73) | 99.1 | MDR-TB | ETB, STR |

* Differences between replicates were only due to low coverage missing genotypes i.e. no differing base calls; ** based on comparing all indels detected by each method; *** based on *TBProfiler;* INH Isoniazid, RIF Rifampicin, STR Streptomycin, ETB Ethambutol, PZA Pyrazinamide, RFB Rifabutin, ETH Ethionamide, AMK Amikacin, CAP Capreomycin, OFX Ofloxacin, MOX Moxifloxacin, PAS Para-aminosalicylic acid, LZ Linezolid, KAN Kanamycin; **bold** - Fluoroquinolone (FLQ) resistance mutation *gyrA* D94A was not found
