# Supplementary material for: The variability and reproducibility of whole genome sequencing technology for detecting resistance to anti-tuberculous drugs
Source: Genome Med. 2016 Dec 22;8:132. doi: 10.1186/s13073-016-0385-x (PMC5178084; doi:10.1186/s13073-016-0385-x)
Supplement: Additional file 9: Table S4. — Mutations that potentially explain drug resistance in the samples. (DOCX 19 kb) [file 13073_2016_385_MOESM9_ESM.docx]

**Additional File 9: Table S4**

**Mutations that potentially explain drug resistance in the samples**

| **Sample –M/XDR-TB** | **INH*** | **RIF*** | **STR^*^** | **ETB^*,^**^£,^ | **PZA^*^** | **ETH^*,**^** | **FLQ^*,£^** | **AMINO^*,£^** | **PAS** |
| --- | --- | --- | --- | --- | --- | --- | --- | --- | --- |
| POR1 –X | *fabG1_pro* C-15T, *inhA* I194T | *rpoB* S450L | *gidB* A80P | *embA_pro* C-16T, *embB* M306V/M423T | *pncA* V125G | *fabG1_pro* C-15T, *inhA* I194T | *gyrA* D94A | *rrs* A1401G | ***thyX G*-4A, *thyX I161T*,** *dfrA-thyA* deletion |
| POR2-M | *inhA* I21V, *katG*  S460N | *rpoB* S450L | - | - | - | *inhA* I21V | - | - | - |
| POR3- X | *fabG1_pro* C-15T, *inhA* S94A | *rpoB* S450L | *rpsL* K43R | *embA_pro* C-12/11AA,  *embB* P397T | **Frameshift mutation**  ***pncA* deletion of nucleotides 437-449** | *fabG1_pro* C-15T, *inhA* S94A | *gyrA* S91P | *tlyA* Ins251TG,  *eis_pro* G-10A | - |
| POR4 –X | *fabG1_pro* C-15T, *inhA* S94A | *rpoB* S450L | *rpsL* K43R | *embB* M306V | *pncA* L120P | *fabG1_pro* C-15T, *inhA* S94A | *gyrA* D94G | *eis_pro* G-10A | - |
| POR5 -M | *fabG1_pro* C-15T, *inhA* I194T | *rpoB* S450L, | *gidB* A80P | *embB* M306V/M423T | *pncA* V125G | *fabG1_pro* C-15T, *inhA* I194T | - | - | - |
| POR6 –X | *fabG1_pro* C-15T, *inhA* I194T | *rpoB* S450L | *gidB* A80P | *embA_pro* C-16T, *embB* M306V | *pncA* V125G | *fabG1_pro* C-15T, *inhA* I194T | *gyrA* D94A | *rrs* A1401G | - |
| POR7 – X | *fabG1_pro* C-15T, *inhA* S94A | *rpoB* S450L, | *rpsL* K43R | *embA_pro* C-12/11AA,  *embB* P397T | *pncA* M1T | *fabG1_pro* C-15T, *inhA* S94A | *gyrA* S91P | *tlyA* Ins251TG,  *eis_pro* G-10A | - |
| POR8 – X | *fabG1_pro* C-15T, *inhA* I194T | *rpoB* S450L | *gidB* A80P | *embA_pro* C-16T, *embB* M306V/M423T | *pncA* V125G | *fabG1_pro* C-15T, *inhA* I194T | *gyrA* D94A | *rrs* A1401G | - |
| POR9 - X | *fabG1_pro* C-15T, *inhA* S94A | *rpoB* S450L | *rpsL* K43R | *embA_pro* C-12/11AA,  *embB* P397T | *pncA* M1T | *fabG1_pro* C-15T, *inhA* S94A | *gyrA* S91P | *tlyA* Ins251TG,  *eis_pro* G-10A | ***folC* S98G** |
| POR10-M | *fabG1_pro* C-15T, *katG* S315T | *rpoB* S450L | *rpsL* K88R | *embB* S297A/M306I | **GG insertion codons 130 and 131 on *pncA*** | *fabG1_pro* C-15T,  *ethA* H281P | - | *eis_pro* C-12T | - |

All mutations on the positive strand; Confirmed using * Sanger sequencing; ** Genotype MTBDR*plus*; ^£^ Genotype MTBDR*sl*, -- *gyrA* S95T, G668A and *gidB* L16R present, but not resistant related; *rpoB* mutations assigned according to *M. tuberculosis* numbering; potentially novel mutations **bolded;** INH isoniazid, RIF rifampicin, STR Streptomycin, ETB Ethambutol, PZA Pyrazinamide, ETH Ethionamide, FLQ fluoroquinolones (Ofloxacin, Moxifloxacin), AMINO Aminoglycosides (Amikacin, Capreomycin, Kanamycin); PAS Para-aminosalicylic acid
